# Deep Learning-Based Location Spoofing Attack Detection and Time-of-Arrival Estimation through Power Received in IoT Networks

**DOI:** 10.3390/s23239606

**Published:** 2023-12-04

**Authors:** Waleed Aldosari

**Affiliations:** Department of Computer Engineering, College of Computer Engineering and Sciences, Prince Sattam bin Abdulaziz University, Al-Kharj 11942, Saudi Arabia; wm.aldosari@psau.edu.sa

**Keywords:** spoofing attack, UAV, IoT, neural network, localization, physical security

## Abstract

In the context of the Internet of Things (IoT), location-based applications have introduced new challenges in terms of location spoofing. With an open and shared wireless medium, a malicious spoofer can impersonate active devices, gain access to the wireless channel, as well as emit or inject signals to mislead IoT nodes and compromise the detection of their location. To address the threat posed by malicious location spoofing attacks, we develop a neural network-based model with single access point (AP) detection capability. In this study, we propose a method for spoofing signal detection and localization by leveraging a feature extraction technique based on a single AP. A neural network model is used to detect the presence of a spoofed unmanned aerial vehicle (UAV) and estimate its time of arrival (ToA). We also introduce a centralized approach to data collection and localization. To evaluate the effectiveness of detection and ToA prediction, multi-layer perceptron (MLP) and long short-term memory (LSTM) neural network models are compared.

## 1. Introduction

The IoT has achieved ubiquitous usage and permeates many facets of our daily lives. IoT networks encompass billions of interconnected devices spanning the internet. Many of these devices possess the ability to sense, process data, and communicate information through various communication channels [1]. An IoT system fulfills four primary functions: the collection of data from the physical environment using sensors, the processing of collected data using embedded systems, the transmission of gathered data through open shared mediums, and—finally—the analysis of the data [2]. A diverse range of devices can be interconnected and communicate over the internet utilizing a multitude of technologies, including wireless sensor networks (WSNs), low-power wide-area networks (LPWANs), Bluetooth, Wi-Fi, long-term evolution (LTE), and a plethora of modern and advanced communication technologies. The IoT has found applications across various domains, including healthcare [3], agriculture [4], smart homes, smart cities, as well as military and civilian sectors [5,6]. In all of these diverse fields, the precise knowledge of the data collection and measurement locations is of paramount importance. Consequently, node localization has emerged as a compelling and open research challenge that is driven by the vulnerability of IoT systems to various attacks, particularly within the context of an open shared communication medium.

Security threats within the IoT, including location spoofing attacks, have escalated to the point of becoming significant and potentially disruptive. In IoT networks, where objects or devices are interconnected for the purpose of accurate measurement, sensing, and task execution, the ability to accurately report the location of collected data is of utmost importance. These location data play a pivotal role in enabling a diverse range of innovative services and functionalities within the IoT ecosystem. Numerous location detection techniques can be employed to identify IoT nodes or objects within IoT systems. GPS is recognized as one of the most widely utilized techniques, providing high-precision coordinates. IoT systems also utilize the ToA, difference time of arrival (DToA), and received signal strength (RSS) to estimate the locations of devices [7,8,9]. While these techniques contribute to improving the accuracy and reliability of IoT node detection and positioning, it is essential to acknowledge their vulnerability to location spoofing attacks and signal spoofing attacks.

Physical layer spoofing attacks can be categorized into passive and active attacks. First, in a passive attack, the attacker receives or listens to the legitimate signal. Eavesdropping is an example of such an attack. The eavesdropper does not propagate or transmit a signal to the target node, making it hard to detect. Second, in an active attack, the attacker intends to transmit unwanted signals to the target, injecting false signals into legitimate signals with the aim of disrupting the communication channel or misleading the IoT device to detect or locate its location. Jamming or signal spoofing attacks are examples of such attacks [10].

This paper primarily focuses on active attacks, specifically addressing one of the most critical IoT location spoofing attacks. In this context, we introduce a novel dataset based on the received power levels of both desired and undesired signals (spoofing signals). The RSS obtained from APs is directly communicated to the target node, whereby it requires location estimation. The research approach involves the development of a neural network model that utilizes this derived dataset to detect spoofed UAVs based on the received signals. Furthermore, we outline a method for locating the spoofer by collaborating with APs, as well as by estimating distances based on the predicted spoofer’s ToA.

This research contributes to the advancement of spoofing signal detection and localization by introducing a feature extraction method based on the received power from a single AP. First, we propose a novel approach, where a single node AP is capable of extracting signal features, which are then fed into a neural network model. This innovative technique allows for efficient spoofing signal detection using a single AP. Additionally, the proposed neural network model has the ability to estimate the spoofer’s ToA based on the received power. This estimated ToA can then be used to estimate the distances from different points, providing valuable information for improving existing localization techniques. By incorporating this estimated distance information, the localization accuracy of current methods can be significantly enhanced. Furthermore, in order to accurately localize the spoofer or detect its location, it is necessary to collect data from multiple APs. This study highlights the importance of a centralized node, where the collected data from multiple APs can be reported and utilized for localization purposes. This centralized approach enables more robust and accurate localizations of the spoofer. The main contributions of this work are as follows:

- Detecting spoofed UAVs and estimating their ToA based on the power received from a single AP in an IoT environment.
- Locating the position of UAVs using the estimated distances between the UAVs and different points, thereby leveraging the predictions.
- Conducting a performance comparison between the MLP and LSTM models, thus resulting in the determination that the MLP model is capable of detecting the presence of a spoofer and estimating its ToA with the received power.

The remainder of this paper is organized as follows. In Section 2, the relevant literature on spoofing attacks in IoT networks is discussed. Section 3 describes the system model. Section 4 provides details on the methodology and materials used, including the two-way protocol and the dataset derived, as well as the feature extraction process. Section 5 explains the comparison between the MLP and LSTM neural network models. Finally, Section 6 and Section 7 present the simulation results and the conclusions of the paper, respectively.

## 2. Literature Review

The physical layer spoofing attack detection method, as presented in [11], relies on a reinforcement learning model. This approach involves the detection of spoofing attacks during the authentication process, which then determines a test threshold through reinforcement learning. Specifically, the method utilizes Q-learning and Dyna-Q techniques, which are tailored for dynamic wireless networks. These techniques are applied to assess the channel states of data packets, thus enabling the detection of spoofing attacks. The overall process can be likened to a zero-sum authentication game that involves both the spoofers and the receiver.

The investigation into detecting GPS spoofing attacks for UAVs presented in [12] focused on a novel approach. In this study, the authors present a GPS spoofing detection method that does not rely on prior knowledge and is based on a derived dataset. Notably, this approach minimizes the need for extensive training data. The proposed model utilizes several key features, including the relative position of the spoofer, variations in the spoofer’s distance, and the angle of arrival of the signal. These features are instrumental in the detection process.

In [13], the authors introduced a technique for estimating the spoofer’s location based on audibility information. This technique leverages a node’s two states: audible and inaudible. By utilizing this audibility information, the problem of location estimation can be re-defined as a stochastic censoring model. Subsequently, the authors derived the maximum likelihood rstimator (MLE) based on the difference time of arrival (DToA) principle. The detection of Wi-Fi spoofing attacks was the subject of investigation in [14]. The authors focused on detecting spoofing in Wi-Fi networks using on-site channel state information (CSI) data. The PHYAlert method presented in this work achieved single-station-based authentication in both stationary and mobile environments.

Detecting and locating spoofing attacks in IoT networks that are based on RSS and the number of connected neighbors (NCN) is discussed in [15]. The authors leverage RSS, delay parameters, and negative acknowledgments to identify spoofing attacks. The analysis employs cluster analysis, which is divided into inter-cluster and intra-cluster detection. In inter-cluster analysis, RSS is employed for detecting and localizing the attacker, while intra-cluster analysis utilizes NCN for localization and detection. Clusters are composed of nodes with similar interests and are maintained by a core node through a status declaration (SD) message or Hello message. Each cluster is further divided into intra-cluster and inter-cluster subsets. To detect spoofing attacks using RSS, the calculated energy between two adjacent nodes is compared to a predefined threshold. If it surpasses the threshold, spoofing detection is triggered. Once the attack is detected, the localization method is initiated through unicasting, as unicasting is unreliable for larger distances in wireless environments. For detecting and localizing spoofing attacks within a cluster, a neighbor detection technique is introduced. In a cluster-based approach, a core node is selected based on various parameters, such as its central position within the group. The prevention and detection of GPS spoofing attacks on UAVs are discussed in [16]. The MLP model processes flight parameters and various features, such as GPS coordinates, position and orientation logs, and system and control status, to generate alarms signaling a GPS spoofing attack. The intrusion detection system is capable of identifying the spoofer when reading the flight parameters collected during one measurement cycle.

The abovementioned algorithms use centralized techniques to detect spoofing attacks in two-dimensional space, as shown in Table 1; however, these approaches increase the communication overhead in IoT networks. In our approach, we aim to detect the presence of spoofed UAVs in a three-dimensional space, as well as predict the spoofer’s ToA using a single node. Furthermore, we collaborated with other nodes to estimate the spoofer’s location.

### 2.1. Neural Networks

Artificial neural networks (ANNs)—or, simply, neural networks (NNs)—represent a specialized subset within the domain of machine learning (ML), forming the fundamental core of deep learning (DL) algorithms [17]. NNs are constructed from information processing units, referred to as neurons. Each neuron within a network is interconnected and possesses associated weights and thresholds, which play a pivotal role during the back-propagation process during training. This mechanism activates neurons and transmits output data to adjacent layers [18]. The weight assigned to each connection signifies the relative importance of a variable in contributing to the output, thereby influencing the data before they are passed to the activation function. Subsequently, the output is compared to a pre-defined threshold; if it surpasses this threshold, the neuron is activated, and the data are relayed to the next layer. In a broader sense, NNs consist of one or more hidden layers in conjunction with input and output layers. Achieving high accuracy and performance hinges on several key factors, including the number of layers, the choice of optimizer, and the quantity of nodes or neurons, all of which require careful consideration when designing a neural network [19].

Various types of NNs exist, including convolutional neural networks (CNNs), recurrent neural networks (RNNs), MLP, LSTM networks, etc. [20]. Each NN type is meticulously engineered to address particular types of tasks and data. Consequently, the judicious selection of an appropriate neural network type for a given task represents a critical consideration in the design and implementation of an artificial intelligence (AI) system. In this research, the objective is to detect spoofed signals and estimate the ToA of the spoofer. To achieve this, the performance of two distinct types of neural networks (NNs) are evaluated and compared, with the aim of achieving high accuracy and improved results. Specifically, we detail the MLP and LSTM models in the following subsection.

### 2.2. Multi-Layer Perceptron (MLP) Network

MLP are feed-forward neural networks with fully connected layers. This architecture is the most common and practical [21]. Typically, an MLP consists of three layers: an input layer, an output layer, and one or more hidden layers. The process begins with the input layer receiving data, followed by the hidden layer processing it and the output layer generating the output data. Each layer is connected to the next layer through weighted edges and biases. During the training process, the weights are adjusted to minimize the difference between the network’s output and the desired output, thus allowing the MLP to learn [22,23].

**Figure 1:**
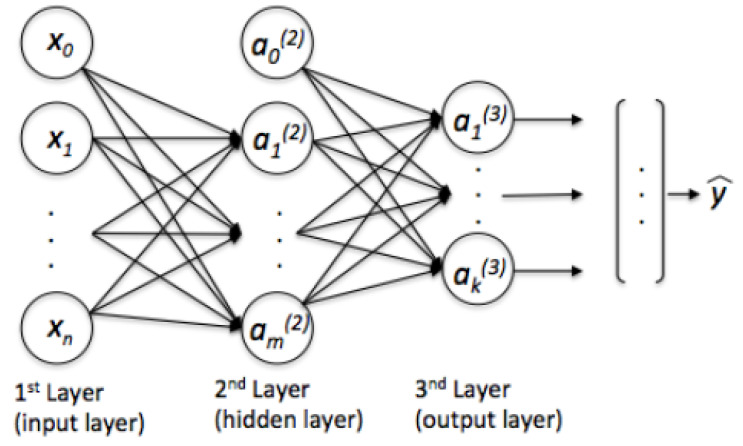
Multilayer perceptron architecture [22].

Activation functions are strategically applied to the outputs of each node in our model to facilitate their transformation into non-linear outputs. The considered MLP model is illustrated in Figure 1, in which the seven scaled features are used as inputs. The hidden layers consist of neurons with rectified linear unit (ReLU) activation functions and a dropout rate of 0.2. The input layer is structured as $X=(x_{0},x_{1},x_{2},\ldots ,x_{n})$, and, through the following ReLU activation function, is used as follows:

(1) f(x)=max(0,x)

The network’s output is computed based on the output of each unit. Specifically, the output of the hidden layer is calculated as follows:

(2) $$h_{i}^{j}=f\sum_{k=1}^{n_{i-1}}w_{k,j}^{i-1}h_{i-1}^{k},\;\;\;\;\;\;i=2,\ldots Nand\;\;j=1,\ldots n_{i}$$

where *h* represents the output of neuron *j* in a hidden layer *i*, *f* denotes the activation function, *w* signifies the weight between neurons *k* in hidden layer i+1, and *n* corresponds to the number of neurons. The network’s output is computed as follows:

(3) $$y_{i}=f\sum_{k=1}^{n_{N}}w_{k,j}^{N}h_{N}^{k},\;\;\;\;\;\;y=y_{1},\ldots ,y_{j},\ldots ,y_{N+1}=F\left(w,x\right)$$

where *Y* represents the vector of the output layer and *F* denotes the transformation function.

### 2.3. Long Short-Term Memory (LSTM)

Recurrent neural networks (RNNs) with LSTM are renowned for their capability to learn and handle long-term data dependencies [24]. They are especially suitable for processing sequential data, as they are explicitly designed to address the challenges posed by long-term dependencies. The network incorporates specialized memory cells that enable it to retain or discard information based on the encountered needs, thus allowing for the storage of data over extended periods of time.

**Figure 2:**
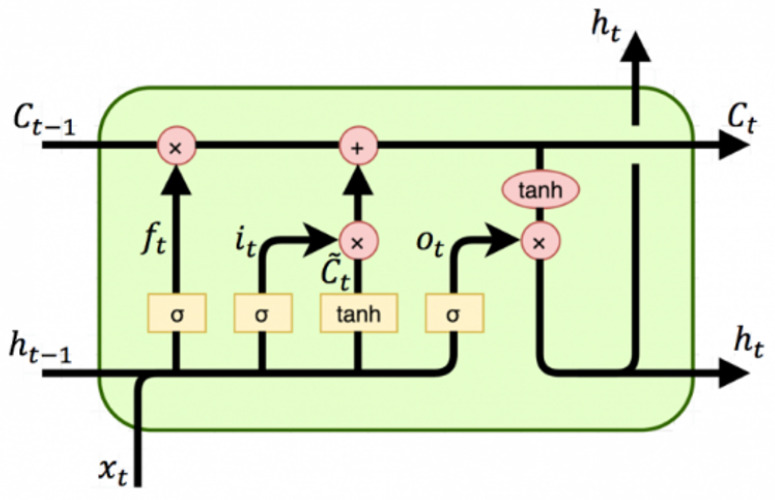
Typical LSTM network [26].

The architecture of an LSTM network is centered around the concept of a memory block, each of which is composed of an input gate, an output gate, and a forget gate, which are represented as *i*, *o*, and *f*, respectively, as visually depicted in Figure 2 [25]. These gates play critical roles in regulating the behavior of the network. The input gate controls the activation of the memory cell, while the output gate governs the activation of the remaining components of the network [26,27]. Within this framework, two vital states are maintained: the cell state, denoted by *C*, and the hidden state *h*. These states are central to the network’s functioning and carry crucial information. Additionally, the architecture incorporates an activation function σ and bias vectors $b_{f}$, $b_{i}$, $b_{C}$, and $b_{o}$, which are integrated into the network. To clarify further, the functions $f_{t}$, $i_{t}$, and $C_{t}$ are described as follows:

(4) $$f_{t}=\sigma \left(W_{f}=\left[h_{t-1},x_{t}\right]+b_{f}\right)$$

(5) $$i_{t}=\sigma \left(W_{i}=\left[h_{t-1},x_{t}\right]+b_{i}\right)$$

(6) $$\bar{C_{t}}=tanh\left(W_{C}=\left[h_{t-1},x_{t}\right]+b_{C}\right)$$

(7) $$C_{t}=f_{t}*C_{t-1}+i_{t}*\bar{C_{t}}$$

(8) $$o_{t}=\sigma \left(W_{o}=\left[h_{t-1},x_{t}\right]+b_{o}\right)$$

(9) $$h_{t}=o_{t}*tanhC_{t}$$

## 3. System Model

**Figure 3:**
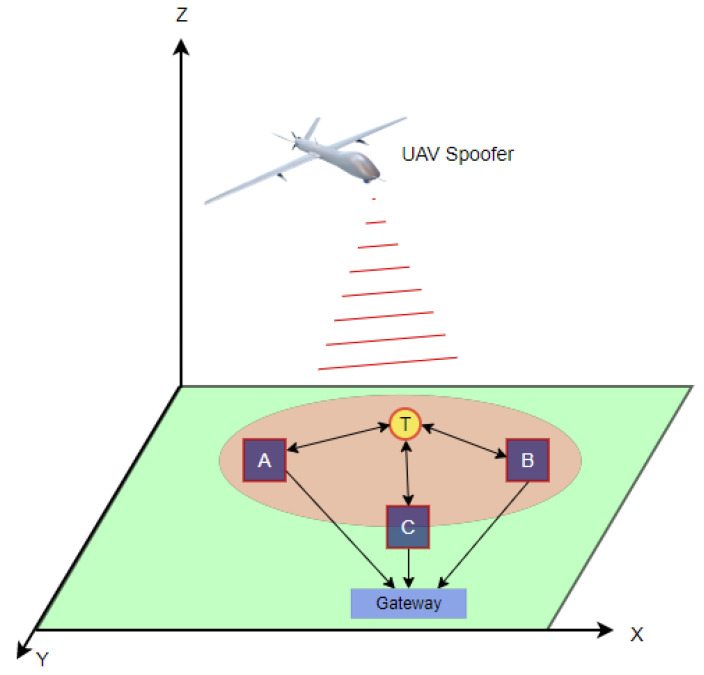
System model.

We considered a wireless network with one target node *T*, whose location is to be estimated, and *N* boundary nodes or APs, whose locations are known. A spoofing device (or attacker) is assumed to be flying over the target area located at position $Xs=[x_{s},y_{s},z_{s}]$. The AP and the target are deployed in a 2-D plane with locations X=[x,y], as shown in Figure 3. The communication channel between the target and AP is modeled using the power loss model [28,29], where the power decreases with increasing distance. A node can communicate with the target node if both have the required signal-to-noise ratio (SNR), which is defined as the difference between the received power and the total noise received by the node $\left(SNR=P_{r}-Noise\right)$ [30]. The SNR is a measure of how much stronger the signal is than the noise, where a higher SNR means that the signal is more likely to be received correctly [31,32].

In the presence of a spoofed UAV, the noise at the receiver increases, leading to a decrease in the SNR. This increases the bit error rate (BER) [33]. Nodes under attack can be classified into three categories based on their SNR: spoofed nodes that may communicate with the AP and have an SNR>γ, edge nodes located at the edge of the spoofed area and have an SNR≈γ, and unhearing nodes with an SNR<γ. The power received at a node is described by the following equation:

(10) $$P_{r}=\frac{P_{t}G_{t}G_{r}}{4\pi d^{2}}+\chi$$

where $P_{r}$ is the received power, $P_{t}$ is the transmitted power, $G_{t}$ is the transmit antenna gain, $G_{r}$ is the receive antenna gain, *d* is the Euclidean distance between the transmitter and receiver $d=\sqrt{{\left(x_{2}-x_{1}\right)}^{2}+{\left(y_{2}-y_{1}\right)}^{2}}$, and χ represents Gaussian noise with a zero mean.

To locate the target node, three anchor nodes, or APs, need to communicate with the target node to collect localization information such as the ToA. This information is then used by a localization algorithm at the central node. However, in the presence of a UAV, the transmit signal from the target node may be deceived by the spoofing signal from the UAV. This can cause a delay in the ToA and a decrease in the SNR, which may result in significant localization errors.

## 4. Materials and Methods

### 4.1. Two-Way Range (TWR) Protocol

**Figure 4:**
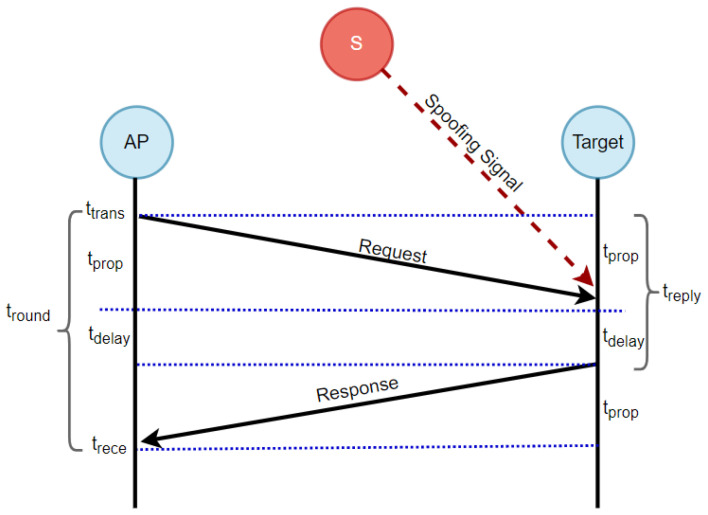
Mechanism of the two-way ranging (TWR) approach.

The TWR protocol is a widely adopted method in IoT networks, particularly in scenarios where time synchronization is unnecessary. Ultra-wideband (UWB) is a radio technology, standardized as IEEE 802.15.4a, which enables the estimation of distance between an AP and a target node [34]. This estimate is obtained by measuring the time it takes for radio frequency (RF) signals to travel between them—known as time-of-flight (ToF)—and subsequently multiplying that time by the speed of light (c) [35]. To estimate the distance between the AP and the target node for localization purposes, the TWR protocol employs the following procedure, as depicted in Figure 4. The AP initiates the process by sending a request packet to the target node at the starting time $\left(t_{trans}\right)$. After a specific delay $\left(t_{delay}\right)$, accounting for processing time and other hardware-related delays (which is assumed to be known by both the AP and the target node), the target node responds to the request message. Subsequently, the AP receives the response message and computes the round trip time ($t_{round}$) to the target node using the following formula [36,37]:

(11) $$t_{round}=2*\left(t_{prop}*t_{trans}\right)+t_{delay}.$$

At the AP, $t_{round}$ is measured by computing the difference between $t_{rece}$ and $t_{trans}$, which is expressed as $t_{round}=t_{rece}-t_{trans}$. Based on $t_{round}$, the AP can compute the propagation time $\left(t_{prop}\right)$ and estimate the distance to the target node using the following equations:

(12) $$t_{prop}=\frac{t_{round}-t_{delay}}{2}$$

(13) $$d=t_{prop}*c$$

In the context of estimating the distance between the AP and the target node, let *d* represent the distance and *c* denote the constant speed of light, which is equal to $3\times 10^{8}m/s$.

### 4.2. Feature Extraction Process

In the scenario where a UAV spoofer is present, the TWR protocol is susceptible to deception, particularly in relation to the received request message and the replay time. The adversary emits undesired signals *S* toward the target, leading to an elevation in the level of noise experienced at the receiver. Consequently, the processing time required to decode the noisy signal is increased. Furthermore, the transmission time for the response message may also be extended due to the utilization of a channel by the adversary or the spoofer. Consequently, this can result in an extension of the sensing time required to identify an available channel. Therefore, the power received at the AP is considered have a significant amount of noise due to the spoofing signal, as presented in the following equation:

(14) $$P_{r}\left(AP\right)=P_{t}-10nlog\left(d\right)+S$$

**Figure 5:**
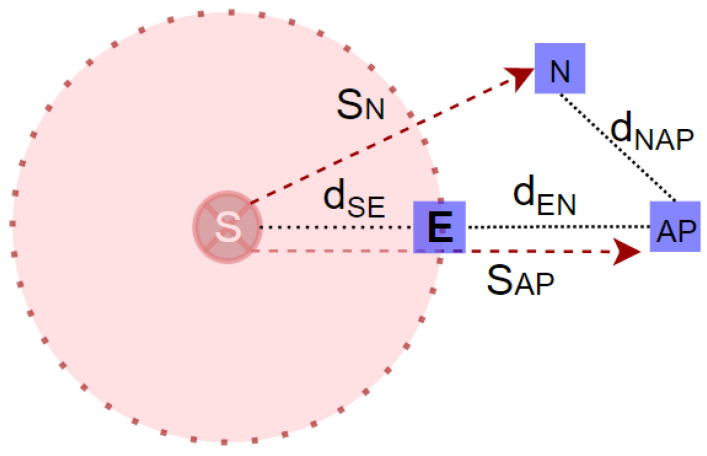
Distance ratio (β) calculation.

In the presence of a spoofing signal *S*, which is considered noise at the receiving side, the SNR at the AP from the target node decreases depending on the position of the UAV spoofer. To estimate the ToA or $\left(t_{prop}\right)$, we utilized the power received at the AP by employing the following approach. The spoofing signal received by the AP, including the received power from the target node, can be presented as follows:

(15) $$P_{r}\left(N,AP\right)=P_{t}\left(N\right)-10nlog\left(d_{NAP}\right)+S$$

where the $P_{r}\left(N,AP\right)$ is the total power received by the AP and S is the spoofing signal. To estimate the $\left(t_{prop}\right)$ between two nodes in presence of a spoofer, we extended the concept of the distance ratio β, which was described in [38], to estimate the ToA at different points. The distance ratio concept is based on the received power and SNR level at the receiving node. When the noise increases, the SNR decreases to reach the threshold value of the system. This means that the AP is located at the edge of the spoofing region, as shown in Figure 5, in which *E* is the edge node, *N* is the node, and AP is the AP. The distance between the spoofer and the AP is denoted by $d_{SN}$, and the distance from the spoofer to the edge node is $d_{SE}$. In this scenario, the edge node is assumed to be a virtual node in order to estimate the ratio of the distance between the spoofer to the edge and from the edge to the AP.

In accordance with Equation (15), we posited the scenario where the AP resides at the boundary of the spoofing region and may receive a spoofing signal while its SNR equals the prescribed system threshold. As a result, the ToA at the AP coincides with the ToA observed at the edge node *E*. In this context, the distances between the spoofer, AP, and edge node remain uniform. For this scenario, the distance ratio is computed as follows:

(16) $$\beta =\frac{d_{SE}}{d_{SN}}\approx 1$$

We also noted a relationship between the distance ratio, the received power, the system threshold value γ, and the noise received (including the spoofing signal), as follows:

(17) $$\beta =10^{\frac{\gamma -P_{r}\left(NAP\right)+S_{AB}}{10\times n}}$$

In our dataset, β serves as the foundational metric for training the deep learning model, thus enabling the detection of the adversary’s presence and its subsequent localization. Here, $d_{SN}$ signifies the distance between the target and the spoofer:

(18) $$d_{SN}=10^{\frac{P_{0}-S_{AB}}{10\times n}}$$

Moreover, the following relationship connects the distance from the edge node to the adversary with β:

(19) $$\left(1-\beta \right)=\left|\frac{d_{EN}}{d_{SN}}\right|$$

Expanding upon Equations (13) and (15), we proceeded to formulate the ToA-based received power as follows:

(20) $$T_{p}\left(NAP\right)=\frac{d_{0}}{c}*10^{\frac{P_{0}-P_{r}\left(NAP\right)}{10\times n}}$$

Hence, the estimate of the ToA at the AP from the spoofer was determined by the overall power received at the AP, as expressed by the following equation:

(21) $$T_{p}\left(SAP\right)=\frac{d_{0}}{c}*10^{\frac{P_{0}-S_{AB}}{10\times n}}$$

**Figure 6:**
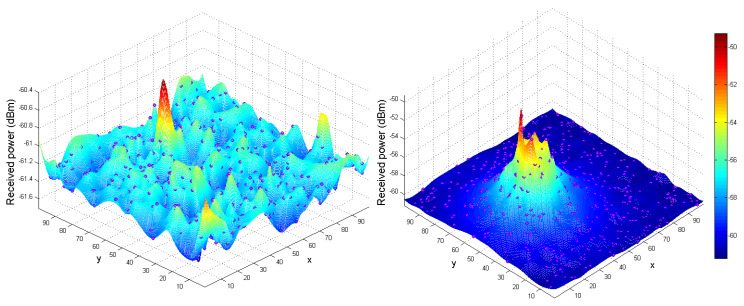
(**Left**) Power received by the AP. (**Right**) The legitimate signal and spoofing signal plotted against the UAV spoofer’s position on the (x,y,z) plane. The purple dots indicate the UAV’s position over 500 steps.

**Figure 7:**
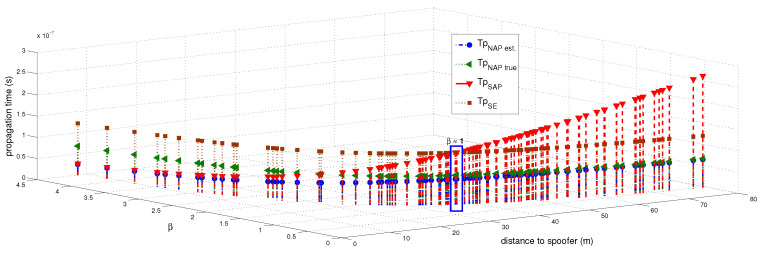
Estimation of the $\left(t_{prop}\right)$ (denoted by $T_{p}$) based on the distance ratio (represented as β) at different points along the spoofer’s trajectory with respect to the AP over 100 steps. Additionally, a scenario where the SNR at the edge node is approximately equal to γ while the distance ratio β is equal to 1 is shown.

Incorporating the spoofer’s signal introduces variations into the ToA values, thus leading to the emergence of a perturbed or noisy ToA measurement. The total power received by the AP, referred to as $S_{AP}$, is illustrated in Figure 6. This measurement encompasses the power received across diverse spatial positions between the AP and the spoofer. Furthermore, it incorporates both the accurate ToA and the ToA for the delayed estimation using β, as demonstrated in Figure 7. Estimation of the ToA at both the edge nodes and from the edge to the AP was accomplished through the utilization of β, as demonstrated by the following equations:

(22) $$T_{p}\left(SE\right)=\frac{d_{SE}}{c}$$

(23) $$T_{p}\left(EAP\right)=\frac{d_{EAP}}{c}$$

The equations above outline the process for estimating ToA values. This process incorporates relative distances and a constant speed of light.

In order to estimate the ToA of the spoofer UAV, as well as to facilitate the localization and detection of the spoofer using a deep learning model, we introduced the detection dataset, as shown in the following section.

### 4.3. Dataset

For this study, we meticulously curated a dataset by harnessing the received power data, alongside the development of a comprehensive system model and scenario simulations using MATLAB. Subsequently, we implemented a deep learning model through Python. Our investigative journey involved a series of experiments and simulations, which were meticulously designed to capture both the authentic and spoofed signals originating from various spoofer locations. The data collection process unfolded as the UAV spoofer executed randomized maneuvers around the designated target node, covering a radius of approximately 100 m. Furthermore, we introduced variations in the spoofer’s transmission power, thus effectively diversifying our dataset. Additional adjustments were made to the transmission power of the nodes, as well as variations in the inter-node distances.

Our dataset encompasses a set of seven distinct input features and two critical output variables, as meticulously delineated in Table 2. These input features are leveraged by our deep learning models to serve a dual purpose: first, to effectively discern the authenticity of the received signals, and, second, to accurately estimate the $\left(t_{prop}\right)$ of the spoofer. Upon the reception of any signal, the AP meticulously extracts the relevant feature values, which are seamlessly integrated into the model’s analytical framework. The dataset used for testing and training the model comprises precisely 2000 samples for testing and 8000 samples for training. This dataset has been thoughtfully curated to ensure balance, whereby it contains an equal number of authentic and spoofed flight times in the scenario.

### 4.4. Pre-Processing and Re-Scaling

Prior to feeding the dataset into the deep learning model, a series of pre-processing steps were meticulously applied to the samples. These pre-processing procedures included the elimination of null and duplicate rows, thus ensuring the integrity of the dataset. Furthermore, encoding techniques were adeptly employed to transform categorical data into numerical representations. In our dataset, a binary classification was established with only two categorical data classes, namely Authentic and Spoofed. To facilitate this categorization, we encoded authentic signals as 0 and spoofed signals as 1, thus effectively converting them into numerical values. Additionally, the ToA values underwent a scaling transformation, whereby they were multiplied by a factor of $10^{7}$. This scaling operation was undertaken to alleviate the presence of extensive fractional values within the dataset, thereby enhancing its suitability for deep learning analysis [39]. Furthermore, the datasets underwent a standardization process using the min-max scaling method. This technique effectively centers the features to have a mean of 0 and a STD of 1, and this achieved using the following equation:

(24) $$Z=\frac{x-x_{min}}{x_{max}-x_{min}}$$

where *x* represents the original data feature, $x_{min}$ corresponds to the feature’s minimum value, and $x_{max}$ denotes the feature’s maximum value. This transformation effectively re-scaled the features within the dataset to span a normalized range of [0,1]. Consequently, the minimum and maximum values of each feature or variable were precisely adjusted to 0 and 1, respectively, as illustrated in Table 3. The dataset features are described in Table 4.

## 5. Proposed Model Architecture

**Figure 8:**
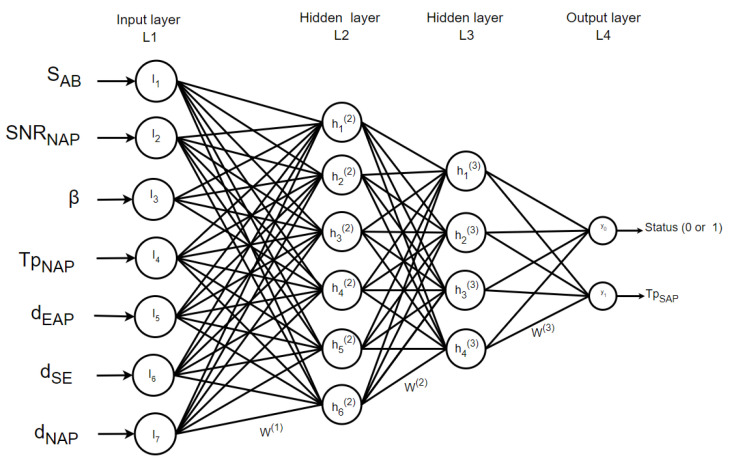
MLP structure.

The MLP was set up and trained with seven input layers, four hidden layers, and two output layers. The hidden layers comprised 164, 64, 32, and 16 units, each utilizing the rectified linear unit (ReLU), as depicted in Figure 8. To prevent overfitting, a dropout rate of 0.2 was applied at the end. The input layer incorporates seven units to feed the extracted features to the model. The output layer consists of two units for detecting spoofing and estimating the ToA with ReLU activation. We utilized the mean square error loss function, Adam optimizer, and ran 800 epochs with a batch size of 25 samples.

**Figure 9:**
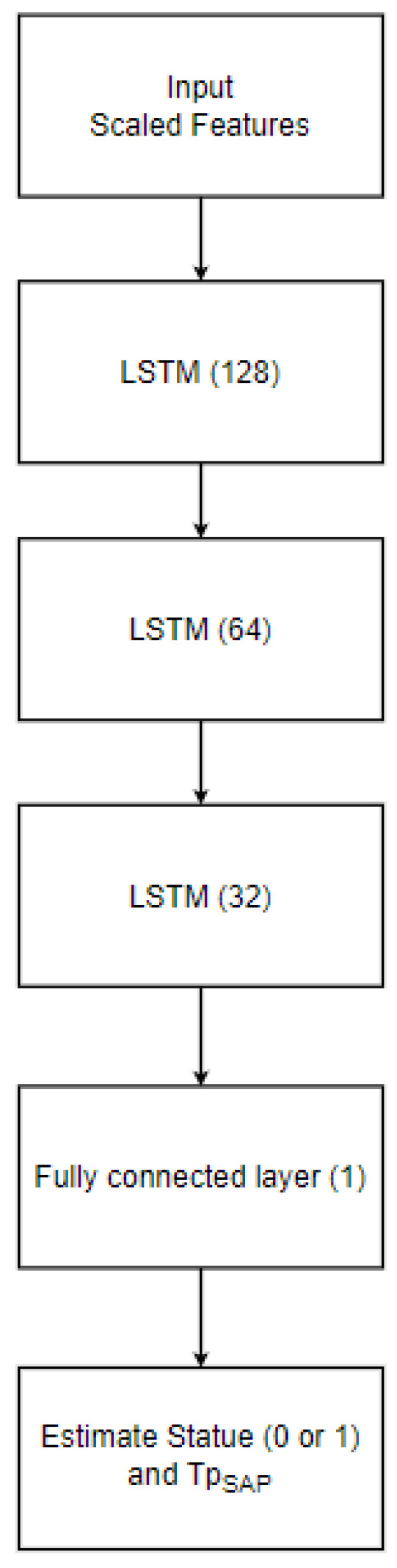
LSTM structure.

**Figure 10:**
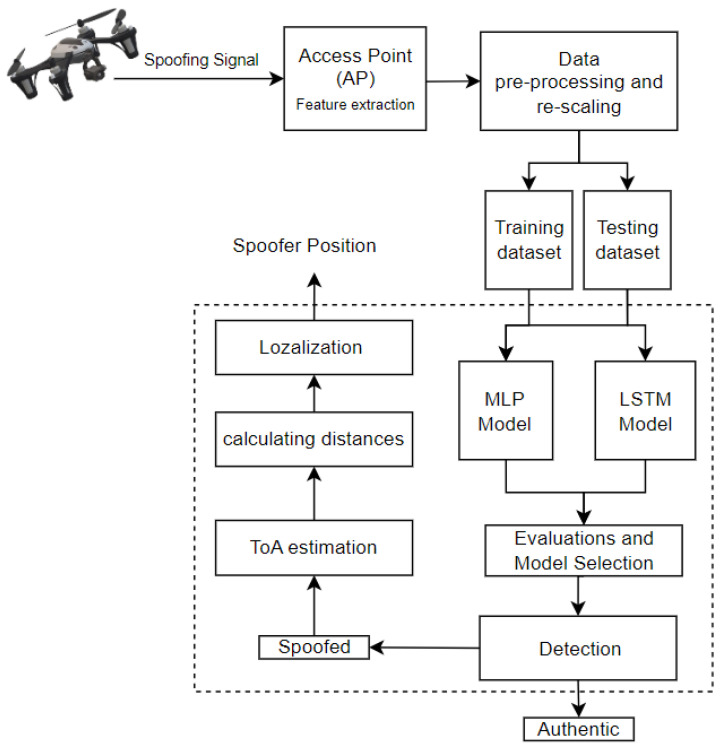
Proposed architecture.

On the other hand, the LSTM consists of three layers for sequence learning in addition to a fully connected layer and a regression layer as the output. The initial layer is composed of 128 hidden units, while the second and third layers consist of 64 and 32 units, respectively. Training was conducted with a mini-batch size of 25 over 800 epochs, as depicted in Figure 9. Throughout each phase, 80% of the data were allocated for training, 10% for testing, and an additional 10% for evaluation purposes. The LSTM employs the same optimizer and loss function as the MLP model. The model structure is designed to detect, estimate, and localize spoofing attacks, as depicted in Figure 10.

### Error Metrics

For our experiment, we employed the root mean square error (RMSE), mean absolute error (MAE), and standard deviation (STD) as evaluation metrics. The equations below illustrate the calculation process, where $\bar{y_{i}}$ represents the estimated value, $y_{i}$ represents the true value, and *n* represents the sample size. RMSE is utilized as a measure of how well the model fits the data, and it is computed through the mean square function. It quantifies the difference between predicted and actual values:

(25) $$RMSE=\sqrt{\frac{1}{n}\sum_{1}^{n}{\left(y_{i}-\bar{y_{i}}\right)}^{2}}$$

Regression models are evaluated using MAE as a metric. This calculation involves taking the average of the absolute errors between the predicted and actual values. The MAE provides a measure of the average magnitude of the errors in the predictions:

(26) $$MAE=\frac{1}{n}\sum_{1}^{n}{\left|y_{i}-{\bar{y}}_{i}\right|}^{2}$$

The R-squared ($R^{2}$) value, also known as the coefficient of determination, is utilized to assess the goodness of fit of a regression model. It measures how closely the data points align with the fitted line. The R-squared value is calculated as the ratio of the residual sum of squares (RSS) to the total sum of squares (TSS):

(27) $$R^{2}=1-\frac{RSS}{TSS}$$

## 6. Simulation and Results

**Figure 11:**
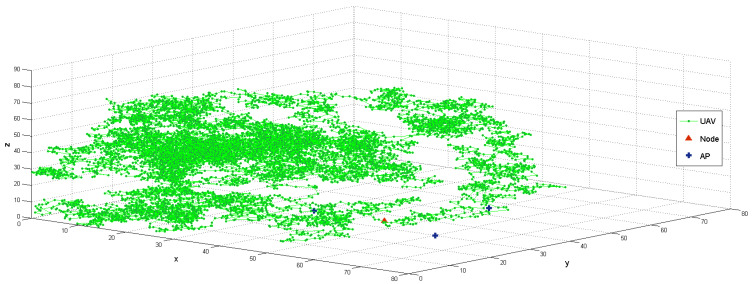
Spoofer UAV hovering around the target area for 10,000 steps. The red triangle represents the target node, the cross line represents the AP, and the green circle depicts the trajectory.

We evaluated the performance of deep neural network models using Python, while the scenarios were simulated using MATLAB. Multiple receivers were randomly positioned for the spoofer UAV as it flew in a three-dimensional space. It started at position (80,50,3), and moved for 10,000 steps while transmitting a spoofing signal at −32dBm. The target and three APs were located at the coordinates (45,35), (30,35), (60,30), and (50,55), respectively, as shown in Figure 11. The transmission power was set to −35dBm for both the target and APs.

The dataset comprises 10,000 samples, consisting of 7 input features and 2 output features. Two deep neural network models—namely MLP and LSTM—were trained on 8000 measurement data points and evaluated on 2000 samples. During model training, the signal status threshold δ, which is for classifying the dataset into authentic and spoofed categories, was determined by taking into account the following factors: the system threshold, the SNR, and the distance from the spoofer.

(28) $$\delta =\left\{\begin{array}{cc} max(\beta_{true})⩽\beta , & \;\;\;\;\;\;Authentic\\ max(\beta_{true})>\beta , & \;\;Spoofed \end{array}\right.$$

**Figure 12:**
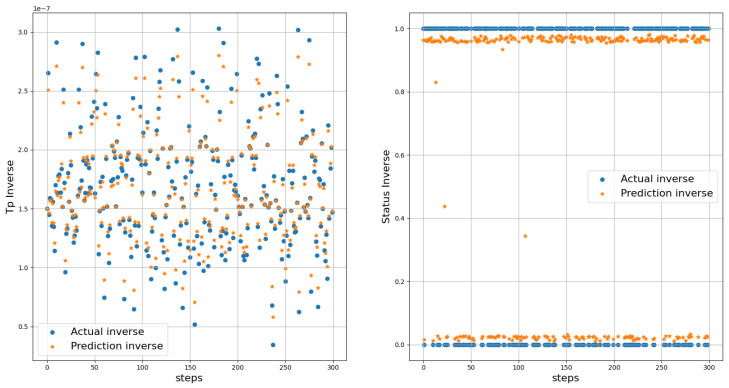
The actual and predicted values of $T_{p}$ and Status. Left figure represents scaled $T_{p}$, while the right figure displays the scaled status.

**Figure 13:**
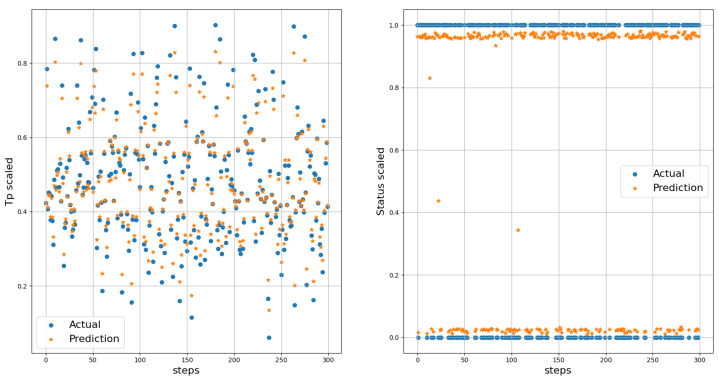
Actual and predicted values of $T_{p}$ and Status. Left figure represents the inverse of $T_{p}$, while the right figure displays the inverse of status.

**Figure 14:**
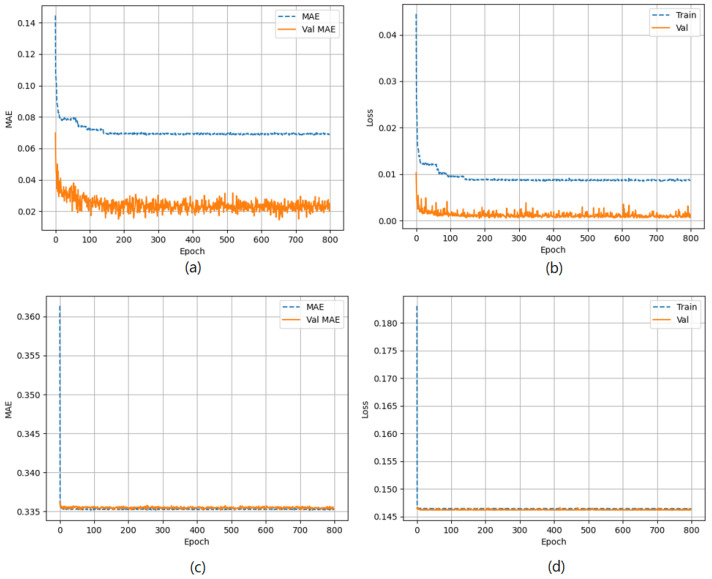
(**a**) The MAE of the MLP model, (**b**) Loss of the MLP model, (**c**) MAE of the LSTM model, and (**d**) Loss of the LSTM model.

The true distance ratio $\beta_{true}$ was evaluated prior to the spoofer initiating signal transmission, where $S_{AP}$ represents the background noise in the absence of the spoofer. According to Equation (16), β is estimated based on the amount of noise and power received by the AP. The results of the performance comparison between the MLP and LSTM models are summarized in Table 5 and Figure 12, Figure 13 and Figure 14. Both the MLP and LSTM models used a patch size of 25 with the Adam optimizer, and a patch size of 125 with the SGD optimizer. Notably, the MLP algorithm outperformed the LSTM model, wherein it exhibited higher accuracy and lower error rates in predicting signal status and spoofer ToA.

The performance of the MLP and LSTM frameworks in spoofing detection is summarized in Table 6, and the results for ToA estimation are presented in Table 7. Both models were trained using a batch size of 25 for 200 epochs when employing the Adam and SGD optimizers. For spoofing detection, the MLP model outperformed the LSTM model significantly in terms of F1 score, accuracy, and precision. Regardless of the optimizer used, either Adam or SGD, the MLP consistently achieved higher scores compared to the LSTM model. The MLP model demonstrated excellent performance with F1 scores close to 1, thus indicating a robust balance between precision and recall. On the other hand, the LSTM model showed lower scores, thereby suggesting an imbalance between precision and recall. For ToA estimation, the MLP model consistently outperformed the LSTM model in both the mean squared error (MSE) and MAE. Regardless of the optimizer, whether Adam or SGD, the MLP achieved lower MSE and MAE values compared to the LSTM. This indicates that the MLP model provides more accurate estimations of the ToA compared to the LSTM model.

### Distance Estimation and Localization

**Figure 15:**
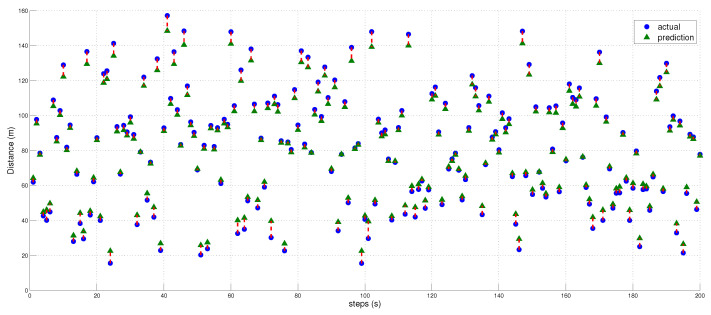
Illustration of the predicted and estimated distances obtained from the MLP model. A comparison between the actual distances and the distances predicted by the model is also shown.

**Figure 16:**
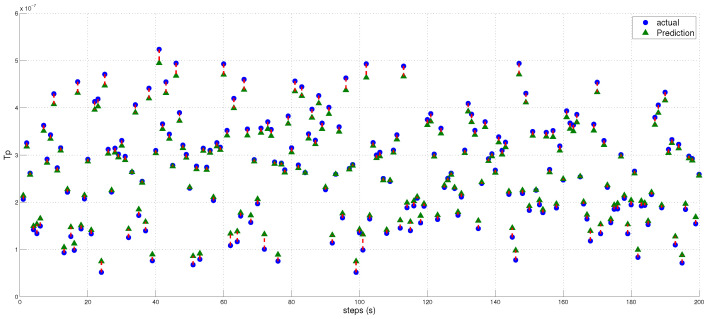
Illustration of the predicted and estimated propagation time (Tp) values by the MLP model. Additionally, it provides a comparison between the actual Tp values and the values predicted by the model.

**Figure 17:**
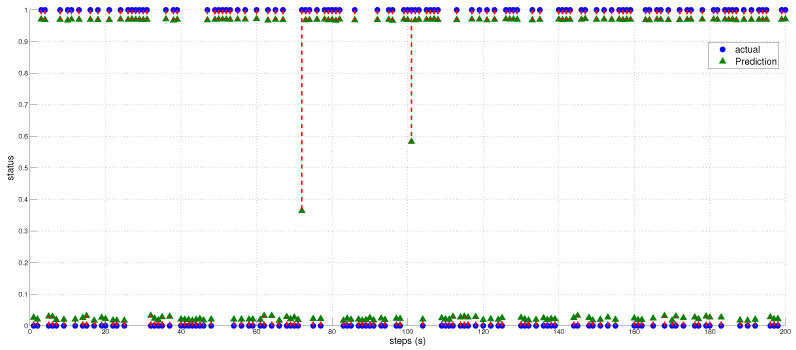
Representation of the predicted and estimated status values obtained from the MLP model. The signal status is indicated, where 0 represents an authentic signal and 1 represents a spoofed signal.

**Figure 18:**
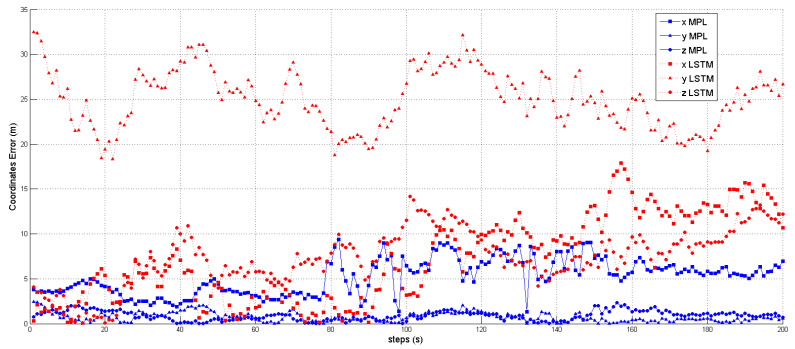
The XYZ coordinates estimated by MLP and LSTM. The coordinates predicted by the LSTM model are represented by the triangle, square, and circle dashed lines, while the solid line depicts the coordinates predicted by the MLP model.

We utilized the predicted inverse ToA to estimate the distance between the spoofer and the edge, as well as that between the edge and the AP using Equations (17)–(23). The estimated distance and the predicted ToA, along with its status, are illustrated in Figure 15, Figure 16 and Figure 17, where 0 signifies authenticity and 1 indicates spoofing. The process of localizing the UAV spoofer involves estimating its location based on predictions from both the LSTM and MLP models, as shown in Figure 18. This localization technique, as presented in [40], utilizes a collaborative approach with APs. The distance information is calculated using Equation (13), wherein the $\left(t_{prop}\right)$ is obtained from the output of the deep learning models. To evaluate the localization performance of both the MLP and LSTM models, a collaborative approach with three APs was employed to determine the spoofer’s location. The evaluation was carried out using key metrics, including the mean square error, STD, and MAE, as summarized in Table 8. The MLP model outperformed the LSTM model significantly in terms of localization errors. With a lower MAE of 2.26 compared to the LSTM’s significantly higher MAE of 13.21, the MLP demonstrated superior accuracy. Similarly, the MLP showed a lower RMSE of 3.06, while the LSTM had a higher RMSE of 15.63. Furthermore, the STD of the MLP, at 2.25, was considerably lower than that of the LSTM, which was at 10.23. These findings underscore the MLP model’s ability to yield more precise localization results with smaller errors when compared to the LSTM model. In contrast, the LSTM model revealed higher errors and higher variability in its predictions.

## 7. Conclusions

In this paper, we presented a novel algorithm designed to detect spoofing signals and accurately predict the ToA of a spoofer in IoT environments. Our algorithm is robust against spoofer attacks and offers significant improvements with respect to the effectiveness and efficiency of location spoofing signal detection and localization systems. The approach begins by estimating the spoofer’s ToA based on the received power at the AP. It then utilizes a feature extraction method using a single AP to detect and predict the spoofer’s ToA. This allows for the estimation of the distances at different points relative to the predicted ToA. Additionally, we collaborated with multiple APs to determine the positions of the spoofed UAVs in 3D space.

The results of our study demonstrate the effectiveness of our proposed model in detecting and localizing spoofing signals. By accurately detecting the presence of spoofing signals and predicting the spoofer’s ToA, we can estimate distances at various points with respect to the predicted ToA. This research contributes to the ongoing efforts to combat location spoofing attacks in IoT-based applications. Our proposed method offers a reliable and robust solution for detecting and localizing spoofing signals, thereby enhancing the security and reliability of location-based services in IoT environments. By addressing the challenges posed by location spoofing attacks, we can ensure the integrity and trustworthiness of IoT systems, thus safeguarding their crucial applications and services. 

## Figures and Tables

**Table 1:** Comparison of the related literature.

Reference	Objectives	Techniques	Evaluation Metrics	Simulator	Application
[11]	Detection	Reinforcement learning	RSS	Software radio peripherals	Indoor environments
[12]	Detection	SVM, deep learning method	Navigation parameters	software package	UAV
[13]	Localization	Location estimation technique	Range, DToA	Monte Carlo	IoT
[14]	Detection	KNN	CSI, OFDM	Commercial off-the-shelf (COTS)	Wi-Fi
[15]	Detection, Prevention	RSS and Number of Connected Neighbors (NCN)	RSS	Network simulator-2	IoT
[16]	Detection	MAVLINK Dataset	Fight system parameters	PX4 autopilot and Gazebo robotics	GPS

**Table 2:** Sample data from the developed dataset.

$S_{\mathrm{AP}}$	${\mathrm{SNR}}_{\mathrm{NAP}}$	β	$T_{p}\left(\mathrm{NAP}\right)$	$d_{\mathrm{NAP}}$	$d_{\mathrm{SE}}$	$d_{\mathrm{EAP}}$	$T_{p}\left(\mathrm{SAP}\right)$	Status
−70.858	18.860	0.202	1.163	34.908	11.110	43.688	1.826	Authentic
−70.859	18.820	0.203	1.163	34.910	11.111	43.442	1.818	Authentic
−70.856	18.805	0.204	1.163	34.901	11.109	43.334	1.814	Authentic
⋮	⋮	⋮	⋮	⋮	⋮	⋮	⋮	⋮
−69.213	3.195	1.231	0.962	28.884	11.108	2.084	0.300	Spoofed
−69.158	3.024	1.255	0.956	28.702	11.110	2.260	0.295	Spoofed
−68.662	1.674	1.466	0.903	27.108	11.111	3.534	0.252	Spoofed

**Table 3:** Sample of scaled data.

$S_{\mathrm{AB}}$	${\mathrm{SNR}}_{\mathrm{NAP}}$	β	$T_{p}\left(\mathrm{NAP}\right)$	$d_{\mathrm{NAP}}$	$d_{\mathrm{SE}}$	$d_{\mathrm{EAP}}$	$T_{p}\left(\mathrm{SAP}\right)$	Status
0.0182	0.6685	0.0807	0.9787	0.9787	0.4904	0.2969	0.3194	0
0.0181	0.6670	0.0813	0.9789	0.9789	0.6168	0.2952	0.3178	0
0.0188	0.6665	0.0815	0.9780	0.9780	0.3642	0.2945	0.3170	0
⋮	⋮	⋮	⋮	⋮	⋮	⋮	⋮	⋮
0.0185	0.6646	0.0823	0.9784	0.9784	0.5627	0.2923	0.3150	1
0.0177	0.6635	0.0827	0.9793	0.9793	0.9064	0.2911	0.3138	1
0.0192	0.6622	0.0833	0.9775	0.9775	0.4269	0.2894	0.3121	1

**Table 4:** Description of features in the developed dataset.

Abbreviation	Description
$S_{AB}$	Total power received by AP
$SNR_{NAP}$	Signal-to-noise ratio at AP
β	Distance ratio
$Tp_{SAP}$	Propagation time between spoofer and AP
$Tp_{NAP}$	Propagation time between target and AP
$d_{NAP}$	Distance between target and AP
$d_{SE}$	Distance between spoofer and edge node
$d_{EAP}$	Distance from edge to AP

**Table 5:** Comparison between MLP and LSTM models.

**Patch Size = 25, Optimizer Adam**						
	**MLP**			**LSTM**		
# of Epoch	RMSE	MAE	std	RMSE	MAE	std
10	0.050	0.508	0.426	0.375	0.512	0.155
100	0.025	0.513	0.430	0.369	0.512	0.155
400	0.024	0.514	0.430	0.370	0.511	0.155
**Patch Size = 25, Optimizer SGD**						
	**MLP**			**LSTM**		
# of Epoch	RMSE	MAE	std	RMSE	MAE	std
10	0.071	0.512	0.424	0.378	0.511	0.155
100	0.036	0.509	0.428	0.378	0.513	0.155
400	0.035	0.512	0.428	0.378	0.512	0.155

**Table 6:** Results for the attack detection.

	Adam Optimizer		SGD Optimizer	
Metrics	MLP	LSTM	MLP	LSTM
Accuracy	99.5%	55.93%	99.98%	55.93%
Precision	100.0%	55.93%	100%	55.93%
Recall	99.04%	100%	99.97%	100%
F1-Score	99.52%	71.74%	99.98%	71.74%

**Table 7:** Results for ToA estimation.

	Adam Optimizer		SGD Optimizer	
Metrics	MLP	LSTM	MLP	LSTM
MSE	0.00047	0.0305	0.00077	0.03049
MAE	0.4929	0.4927	0.4925	0.4944

**Table 8:** Localization error results.

	MAE	Std	RMSE
**MLP**	2.26	2.25	3.06
**LSTM**	13.21	10.23	15.63

## Data Availability

Data are contained within the article.
